# Long-term outcome of biologic graft: a case report

**DOI:** 10.1186/1752-1947-8-255

**Published:** 2014-07-17

**Authors:** Kim-Phung Nguyen, Veronica Zotos, Eddy C Hsueh

**Affiliations:** 1 Division of General Surgery, Department of Surgery, Saint Louis University, 3635 Vista at Grand Blvd, St. Louis, MO 63110, USA; 2 St. Anthony’s Medical Center, 10010 Kennerly Road, St. Louis, MO 63128, USA

**Keywords:** Abdominal wall reconstruction, Biologic graft, Hernia

## Abstract

**Introduction:**

Biologic grafts have been shown to support tissue regeneration in various animal models. Very few reports in the literature exist to show tissue remodeling in patients after placement of a biologic graft.

**Case presentation:**

We report the case of a 69-year-old Caucasian man with a history of small bowel carcinoid resection and concurrent recurrent ventral hernia repair with component separation and underlay biologic graft placement who underwent re-operation for metastatic carcinoid tumor to his liver. Complete incorporation of the biologic graft was observed. Tissue analysis of the incised midline fascia revealed tissue remodeling at the site of the previous abdominal wall defect.

**Conclusion:**

Placement of a biologic graft in ventral hernia repair supports tissue regeneration similar to that previously reported in animal models.

## Introduction

The use of biologic graft materials in complex abdominal wall reconstruction over the last several decades has significantly increased, especially in contaminated cases where synthetic mesh is relatively contraindicated. Two important potential complications after complex abdominal wall reconstruction are recurrence and infection. Several studies have shown that although recurrence rates are slightly lower in patients who have received synthetic mesh than in those who have received biologic graft material, the complication rates, especially the incidence of infection, are higher [1]. The morbidity associated with an infected synthetic mesh is greater than an infected biologic graft because management of an infected synthetic mesh requires antibiotics and removal of the foreign material, whereas infection of a biologic graft can be managed with antibiotics and wound care and does not typically require graft removal [2].

A recent study evaluating biologic graft biopsies obtained during re-exploration for ventral hernia recurrence revealed that the duration of time the biologic graft had been implanted was directly correlated with the degree of constructive remodeling. The human dermis scaffolds that had been implanted longer showed decreased numbers of inflammatory cells and fibrous encapsulation, increased extracellular matrix deposition and neovascularization, increased cellular penetration to the center of the biopsy, and nearly complete scaffold degradation [3].

Animal studies utilizing either porcine or human acellular dermal matrices showed that after several weeks, the tensile strength measurements of the abdominal wall at the biologic graft-to-musculofascia interface were not less, and in some cases were greater, than the adjacent unwounded fascia [4-6]. In addition, histological and immunohistochemical analysis demonstrated remodeling into fascia-like tissue with gene expression that was similar to the native fascia. For example, the surface of the biologic graft placed apposite to the bowel exhibited cellularity consistent with peritoneum and collagen fiber architecture similar to the alignment of native fascia [4-6]. This suggests an active regenerative process that allows for restoration of abdominal wall function. Despite the increasing popularity of biologic grafts in hernia repair, long-term histological evidence of biologic graft incorporation in humans is scarce as healed surgical sites are rarely revisited.

## Case presentation

**Figure 1:**
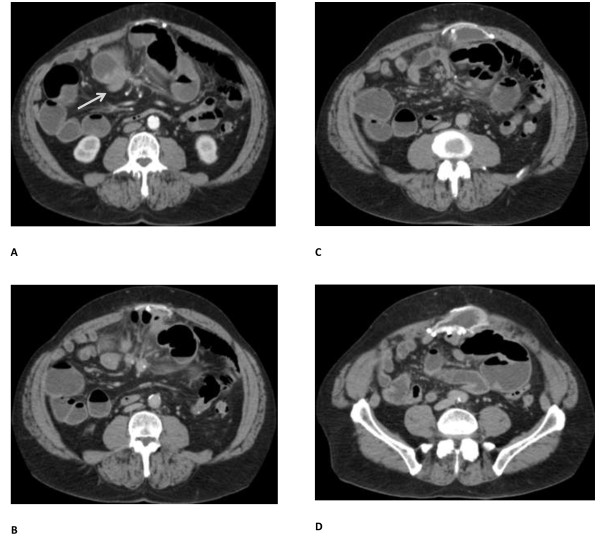
**Cross-sectional computed tomography images of patient pre-biologic graft placement. A**. Arrow indicates the carcinoid tumor. Presence of synthetic mesh is also shown. **B**-**D**. Additional caudad images demonstrating the presence of three synthetic meshes, mesh-associated seroma, and hernia recurrence due to separation of native fascia and mesh interface.

A 69-year-old Caucasian man presented with recurrent metastatic carcinoid tumor to his liver. His past surgical history included gunshot wounds to his abdomen, resulting in an exploratory laparotomy in 1979. Four years later, a ventral hernia developed that was repaired primarily. His hernia recurred another 5 years later and was repaired with a polypropylene mesh.In 2009, he developed gastrointestinal symptoms of intermittent bloating, epigastric pain, abdominal cramps, and alternating episodes of diarrhea and constipation. With increased frequency and severity of symptoms, he subsequently developed a third recurrence of his ventral hernia in late 2010. A small bowel mass was visualized on a computed tomography scan in 2011 along with the recurrent ventral hernia (Figure 1A-D). This prompted serologic testing and radionuclide scanning studies that lead to a diagnosis of carcinoid tumor. An octreotide scan revealed uptake in his distal small bowel as well as the dome of his liver suspicious for metastatic carcinoid tumor with involvement of his liver.

He underwent exploratory laparotomy for the metastatic carcinoid tumor in March 2011. A 5cm right flank ventral hernia located at the edge of the polypropylene mesh was found. Extensive adhesiolysis, small bowel resection with primary anastomosis, cholecystectomy, intra-operative ultrasound-guided microwave ablation of segment 8 liver lesion (pathology positive for metastatic neuroendocrine carcinoma), and a ventral hernia repair were performed.

**Figure 2:**
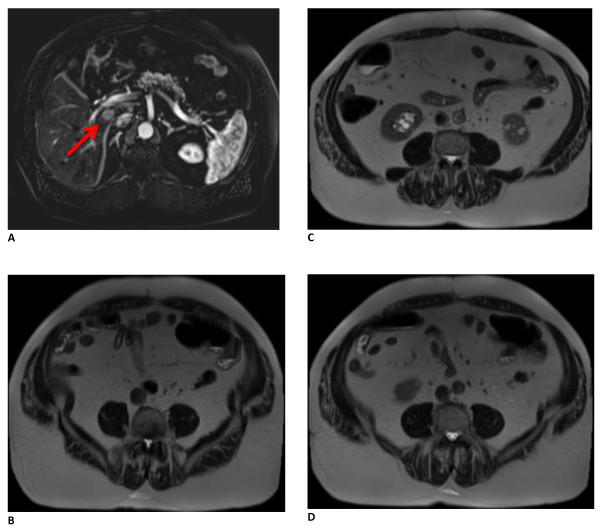
**Cross-sectional magnetic resonance images of patient 30 months after biologic graft placement. A**. Arrow indicates the recurrent carcinoid tumor in the liver. **B**-**D**. Additional caudad images demonstrating abdominal wall integrity.

**Figure 3:**
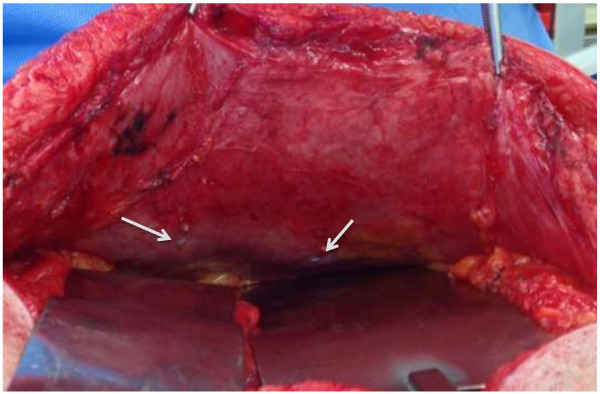
**Intraoperative photo of fascia where underlay biologic graft was placed.** Intraoperative photo of fascia in the region of previously placed biologic graft with previously placed transfascial O Ethibond sutures (arrows). There was no visual evidence of graft material.

**Figure 4:**
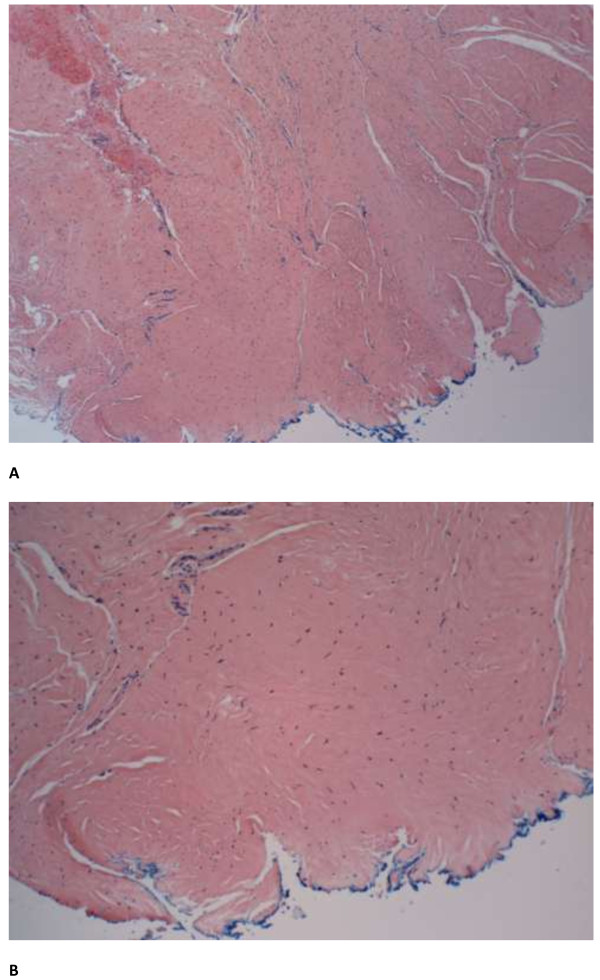
**Histology analysis of midline fascia edge sampling.** Vascularized dense connective tissue at previously placed biologic graft site. Blue ink indicates peritoneal edge of the fascia. **A**. 100× magnification. **B**. 200× magnification.

During the ventral hernia repair, the previously placed polypropylene mesh was removed. The hernia sac was excised and component separation consisting of a bilateral rectus abdominis muscle advancement flap was performed. His abdominal wall was reinforced with a 20cm×20cm non-cross-linked biologic hernia repair graft underlay (Biodesign^®^, Cook Medical, Bloomington, IN, USA) and secured with transfascial O Ethibond sutures (Ethicon, Blue Ash, OH, USA). The fascia edges were approximated primarily over the biologic graft with #1 polydioxanone (PDS) and drains were placed in the subcutaneous layers. He recovered uneventfully.Thirty months after the carcinoid resection and liver tumor ablation, a repeat surveillance octreotide scan revealed a suspicious 1.9cm lesion on segment 1 of his liver that was confirmed with magnetic resonance imaging. Good fascial approximation at the site of the prior recurrent ventral hernia repair was also noted (Figure 2). He underwent surgery for recurrent metastatic carcinoid tumor ablation in October 2013. On visual inspection, there was no clear demarcation of the previously placed biologic graft except for the presence of the permanent transfascial Ethibond sutures (Figure 3). His native fascia was observed. Tissue biopsies of the incised midline fascia were obtained. Histology of the resected section revealed vascularized dense connective tissue (Figure 4).

## Discussion

Abdominal wall reconstruction with synthetic mesh or biologic graft reinforcement has reduced hernia recurrence as compared to primary repair. Mesh positioning can include onlay, underlay, interposition, or retrorectus placement. Underlay or retrorectus placement has been associated with lower recurrence rates [7].

Polyester, polypropylene, and polytetrafluoroethylene (PTFE) are several types of materials used in prosthetic mesh. The mechanism by which prosthetic mesh strengthens hernia repair is by inciting an intense inflammatory response to the foreign body with resultant development of a strong scar plate. The inflammatory changes around the alloplastic material used in abdominal wall defect repair can persist for many years [8]. This intense inflammatory response and subsequent scar plate formation can contribute to increased stiffness of the abdominal wall and shrinkage of the mesh. The use of “lightweight” mesh to decrease this inflammatory response has resulted in improved abdominal wall compliance and less mesh shrinkage [9]. In a clean abdomen without bacterial contamination, prosthetic mesh repair remains the standard of care for optimal long-term results.

The various types of biologic graft materials consist of a decellularized collagen matrix derived from either porcine, bovine or human sources. Typically obtained from either dermal or non-dermal sources these graft materials have undergone significant processing to remove the cells and antigenic components, resulting in a highly purified collagen architecture and surrounding extracellular matrix. Depending on the graft, the resulting product may or may not be cross-linked [10]. The theory behind the use of biologic grafts is that they provide scaffolding for cells and new blood vessels to infiltrate, support tissue regeneration, and can be used in the face of infection where synthetic materials are relatively contraindicated.

Common hernia mesh implant complications include recurrence, infection, seroma formation, integumentary necrosis, chronically draining sinuses, enterocutaneous fistulas, and intestinal obstruction [11]. Both classical synthetic mesh materials and the more modern acellular biologic graft materials may lead to post-hernia repair complications. In particular, a review of the literature has shown that postoperative complications involving synthetic meshes are more likely to lead to mesh extrusion and extended comorbid complications, such as a chronic inflammatory response resulting in chronic pain, sensation of feeling the mesh, and stiffness of the abdominal wall with loss of compliance [12]. These complications can necessitate removal of the mesh to fully resolve. However, complications involving many of the biologic graft implants, such as infection, are treated more conservatively [13].

Although a retrospective study assessing single-stage ventral hernia repairs with lightweight polypropylene mesh implants in clean-contaminated and contaminated cases has shown some favorable results, re-operation was 12% and the incidence of surgical site occurrences (surgical site infection, wound dehiscence, or wound breakdown) was 30% [14]. A systematic review of several retrospective studies utilizing biologic grafts showed an overall recurrence rate of 13.8%, which increased to 23.1% in contaminated/dirty repairs [15]. Biologic tissue matrices are commonly used in contaminated or infected surgical fields for one-stage repair with little to no subsequent graft removal, but hernia recurrence over a 5-year period may be greater than 50% [16]. Therefore, the durability of biologic grafts for single-stage repair in contaminated settings needs further evaluation [17]. Further prospective, randomized trials need to be conducted before practice guidelines are established.

Animal studies of various biologic graft materials on physicomechanical properties [18], immunological responses [19], neovascularization and collagen formation [20], and other correlates of tissue repair have been widely reported. The purported mechanism of hernia defect repair with biologic grafts is by integration of the biologic graft as a scaffold for tissue remodeling via neovascularization and tissue regeneration. However, histologic evidence of tissue remodeling after biologic graft placement is lacking in humans, as few patients undergo postoperative tissue analysis to verify the degree of graft incorporation. To demonstrate clinically the claim of tissue remodeling and graft incorporation as suggested by the animal data would require serial biopsies over time and would not be ethically feasible in any patient recovering uneventfully from complex hernia repair. The current case provides clinical evidence that long-term abdominal wall integrity was achieved in a patient with a third recurrence of ventral hernia following component separation repair using a biologic graft as a temporary scaffold.

## Conclusions

Several animal studies have shown that biologic grafts induce the expression of extracellular matrix components, remodeling enzymes, and inflammatory cytokines, and promote fibroblast infiltration and migration into the matrices. Our current observation suggests that tissue regeneration in humans may mirror that of the animal model. Improved tissue regeneration with implantation of biologic graft materials in animals may also be seen in humans.

## Consent

Written informed consent was obtained from the patient for publication of this case report and any accompanying images. A copy of the written consent is available for review by the Editor-in-Chief of this journal.

## Abbreviations

CT scan: Computed tomography; MRI: Magnetic resonance imaging.

## Competing interests

The authors declare that they have no competing interests.

## Authors’ contributions

EH performed the clinical follow-up of the patient. EH performed the surgery. KN and EH have made substantial contributions to conception and design, or acquisition of data, or analysis and interpretation of data. Furthermore, all authors have been involved in revising the manuscript critically for important intellectual content, and have read and approved the final manuscript. VZ provided the histologic pictures and analysis.

## References

[B1] Ghazi B Deigni O Yezhelyev M Losken A Current options in the management of complex abdominal wall defects Ann Plast Surg 2013 66 5 488 492 10.1097/SAP.0b013e31820d18db 21372667

[B2] Collage RD Rosengart MR Abdominal wall infections with *in situ* mesh Surg Infect (Larchmt) 2010 11 3 311 318 10.1089/sur.2010.029 20583867

[B3] Cavallo JA Roma AA Jasielec MS Ousley J Creamer J Pichert MD Baalman S Frisella MM Matthews BD Deeken CR Remodeling characteristics and collagen distribution in biological scaffold materials explanted from human subjects after abdominal soft tissue reconstruction: An analysis of scaffold remodeling characteristics by patient risk factors and surgical site classifications Ann Surg 2013 [Epub ahead of print] 10.1097/SLA.0000000000000471 PMC4072753 24374547

[B4] Butler CE Burns NK Campbell KT Mathur AB Jaffari MV Rios CN Comparison of cross-linked and non-cross-linked porcine acellular dermal matrices for ventral hernia repair J Am Coll Surg 2010 211 3 368 376 10.1016/j.jamcollsurg.2010.04.024 20800194

[B5] Xu H Wan H Sandor M Qi S Ervin F Harper JR Silverman RP McQuillan DJ Host response to human acellular dermal matrix transplantation in a primate model of abdominal wall repair Tissue Eng Part A 2008 14 12 2009 2019 10.1089/ten.tea.2007.0316 18593339

[B6] de Castro Bras LE Shurey S Sibbons PD Evaluation of crosslinked and non-crosslinked biologic prostheses for abdomi- nal hernia repair Hernia 2012 16 1 77 89 10.1007/s10029-011-0859-0 21805341 PMC3266498

[B7] Albino FP Patel KM Nahabedian MY Sosin M Attinger CE Bhanot P Does mesh location matter in abdominal wall reconstruction? A systematic review of the literature and a summary of recommendations Plast Reconstr Surg 2013 132 1295 1304 10.1097/PRS.0b013e3182a4c393 24165612

[B8] Klinge U Klosterhalfen B Muller M Schumpelick V Foreign body reaction to meshes used for the repair of abdominal wall hernias Eur J Surg 1999 165 665 673 10.1080/11024159950189726 10452261

[B9] Cobb WS Kercher KW Heniford BT The argument for lightweight polypropylene mesh in hernia repair Surg Innov 2005 12 1 63 69 10.1177/155335060501200109 15846448

[B10] Cornwell KG Landsman A James KS Extracellular matrix biomaterials for soft tissue repair Clin Podiatr Med Surg 2009 26 507 523 10.1016/j.cpm.2009.08.001 19778685

[B11] Leber GE Garb JL Alexander AI Reed WP Long-term complications associated with prosthetic repair of incisional hernias Arch Surg 1998 133 378 382 10.1001/archsurg.133.4.378 9565117

[B12] Bellows CF Shadduck PP Helton WS Fitzgibbons RJ The design of an industry-sponsored randomized controlled trial to compare synthetic mesh versus biologic mesh for inguinal hernia repair Hernia 2011 15 325 332 10.1007/s10029-010-0773-x 21222008

[B13] Patel KM Bhanot P Complications of acellular dermal matrices in abdominal wall reconstruction Plast Reconstr Surg 2012 130 216S 224S 23096976 10.1097/PRS.0b013e318262e186

[B14] Carbonell AM Criss CN Cobb WS Novitsky YW Rosen MJ Outcomes of synthetic mesh in contaminated ventral hernia repairs J Am Coll Surg 2013 217 6 991 998 10.1016/j.jamcollsurg.2013.07.382 24045140

[B15] Slater NJ van der Kolk M Hendriks T van Goor H Bleichrodt RP Biologic grafts for ventral hernia repair: a systematic review Am J Surg 2013 205 2 220 230 10.1016/j.amjsurg.2012.05.028 23200988

[B16] Kissane NA Itani KM A decade of ventral incisional hernia repairs with biologic acellular dermal matrix: what have we learned? Plast Reconstr Surg 2013 130 194S 202S 10.1097/PRS.0b013e318265a5ec 23096971

[B17] Rosen MJ Krpata DM Ermlich B Blatnik JA A 5-year clinical experience with single-staged repairs of infected and contaminated abdominal wall defects utilizing biologic mesh Ann Surg 2013 257 6 991 996 10.1097/SLA.0b013e3182849871 23426340

[B18] Deeken CR Eliason BJ Pichert MD Grant SA Frisella MM Matthews BD Differentiation of biologic scaffold materials through physicomechanical, thermal, and enzymatic degradation techniques Ann Surg 2012 255 595 604 10.1097/SLA.0b013e3182445341 22314328

[B19] Orenstein SB Qiao Y Klueh U Kreutzer DL Novitsky YW Activation of human mononuclear cells by porcine biologic meshes *in vitro* Hernia 2010 14 401 407 10.1007/s10029-010-0634-7 20145965

[B20] Badylak S Kokini K Tullius B Simmons-Byrd A Morff R Morphologic study of small intestinal submucosa as a body wall repair device J Surg Res 2002 103 190 202 10.1006/jsre.2001.6349 11922734

